# RETRACTED ARTICLE: Direct healthcare costs of spinal disorders in Brazil

**DOI:** 10.1007/s00038-018-1099-1

**Published:** 2018-04-12

**Authors:** Rodrigo Luiz Carregaro, Everton Nunes da Silva, Maurits van Tulder

**Affiliations:** 1 School of Physical Therapy, Universidade de Brasília (UnB), Campus UnB Ceilândia, Centro Metropolitano, conjunto A, lote 01, Brasília, DF CEP 72220-275 Brazil; 2 Department of Health Sciences, Faculty of Science, Vrije Universiteit Amsterdam, Amsterdam, The Netherlands; 3 School of Collective Health, Universidade de Brasília (UnB), Campus UnB Ceilândia, Brasília, Brazil

This article has been retracted as it was inadvertently moved to production before final acceptance, the Journal and the Publisher apologise for this error. The authors have been given the opportunity to resubmit the manuscript to the Journal to complete the peer review process. The authors agree to this retraction. The online version of this article contains the full text of the retracted article as electronic supplementary material.

## Electronic supplementary material

Below is the link to the electronic supplementary material.

Supplementary material 1 (PDF 79 kb)

Supplementary material 2 (PDF 441 kb)

