# Supplementary material for: RETRACTED ARTICLE: Direct healthcare costs of spinal disorders in Brazil
Source: Int J Public Health. 2018 Apr 12;64(6):975. doi: 10.1007/s00038-018-1099-1 (PMC6614539; doi:10.1007/s00038-018-1099-1)
Supplement: Supplementary file 1 — Supplementary material 1 (PDF 79 kb) [file 38_2018_1099_MOESM1_ESM.pdf]

Direct healthcare costs of spinal disorders in Brazil

International Journal of Public Health

Rodrigo Luiz Carregaro[1,2,*]; Everton Nunes da Silva[3]; Maurits van Tulder[2].

1. School of Physical Therapy, Universidade de Brasília (UnB), Campus UnB Ceilândia, Brasilia, Brazil.
2. Department of Health Sciences, Faculty of Science, Vrije Universiteit Amsterdam, Amsterdam, The Netherlands.
3. School of Collective Health, Universidade de Brasília (UnB), Campus UnB Ceilândia, Brasilia, Brazil.

* Corresponding author: rodrigocarregaro@unb.br

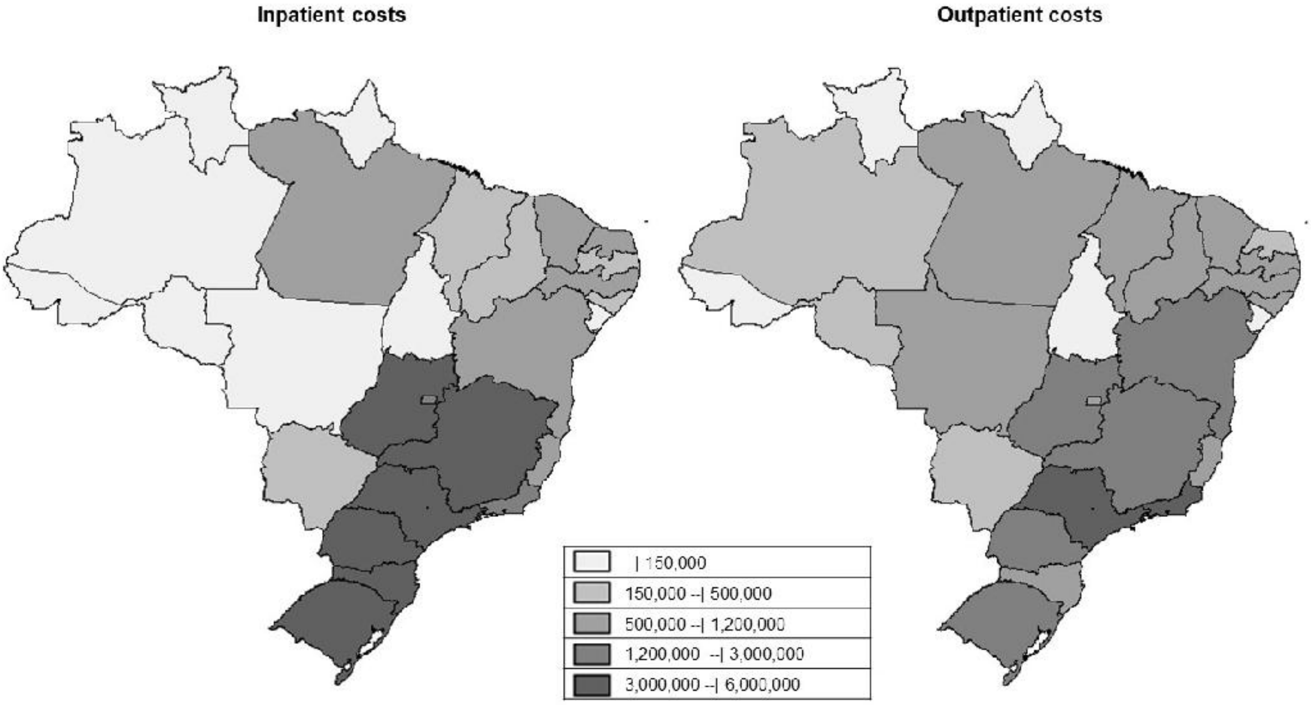

Fig. 1 Distribution of the total healthcare cost (inpatient and outpatient) over the Brazilian states in 2016. The grey boxes represent ranges of expenditure, and values are presented in US$

Table 1. Most common procedures reported as reason for hospital admissions in 2016.

Nature of procedure	Quantity	Most used	Quantity
Diagnostic	13,328	Tissue Biopsy	13,287
Clinical	310,163	Treatment of complications after surgical or clinical procedures	97,042
		Drug treatment for severe pain	85,825
		Clinical treatment of myelitis/myelopathies	24,046
		Treatment of patients undergoing long-term care (musculoskeletal diseases)	20,654
		Diagnosis and/or emergency care in medical clinic	19,624
Surgery	776,316	Arthrodesis	115,148
		Treatment with multiple surgeries	106,997
		Discectomy	26,200
		Arthroplasty (revision or reconstruction of the hip)	26,148
		Surgical treatment of spinal deformity	25,228
Organ, tissue and cell transplants	1,184	Intercurrent treatment post-transplant	1,184
**Total Amount (in 2016)**	**1,100,991**	**Total**	**561,383**

Table 2. Most common procedures/services adopted during inpatient care in 2016.

Nature of procedure	Quantity	Most used	Quantity
Physiotherapy	102,374	Physiotherapy: neurology	42,114
		Physiotherapy: cardiorespiratory	27,783
		Physiotherapy: musculoskeletal	20,114
		Physiotherapy: oncology	8,551
Diagnostic	39,289	Hemotherapy diagnostic	15,268
		Computed Tomography	11,393
		Magnetic Resonance Imaging	5,142
		Pathological examinations	3,364
		Ultrasonography	2,585
Surgery	4,153	Neurosurgery: trauma and developmental disorders	1,842
		Neurosurgery: spine and peripheral nerves	984
		Oncology surgery	829
		Neurosurgery: pain treatment	190
**Total Amount (in 2016)**	**145,816**	**Total**	**140,159**
