# Supplementary material for: RETRACTED ARTICLE: Direct healthcare costs of spinal disorders in Brazil
Source: Int J Public Health. 2018 Apr 12;64(6):975. doi: 10.1007/s00038-018-1099-1 (PMC6614539; doi:10.1007/s00038-018-1099-1)
Supplement: Supplementary file 2 — Supplementary material 2 (PDF 441 kb) [file 38_2018_1099_MOESM2_ESM.pdf]

International Journal of Public Health
https://doi.org/10.1007/s00038-018-1099-1

SSPH+ SWISS SCHOOL OF PUBLIC HEALTH +

ORIGINAL ARTICLE

CrossMark

RETRACTED ARTICLE

Direct healthcare costs of spinal disorders in Brazil

Rodrigo Luiz Carregaro[1,2] · Everton Nunes da Silva[3] · Maurits van Tulder[2]

Received: 26 March 2018 / Accepted: 5 April 2018
© The Author(s) 2018

Abstract

Objectives To investigate the direct healthcare costs of spinal disorders in Brazil, over 2016.

Methods Prevalence-based cost-of-illness study, top-down approach and public healthcare system's perspective. International Classification of Diseases codes related to spinal disorders were included. The following costs were obtained: (1) hospitalization; medical professional service costs; intensive care unit costs; companion daily stay; (2) outpatient (services/procedures). Data were analyzed descriptively and costs presented in US$.

Results The healthcare system spent US$ 71.4 million and inpatient care represented 58%. The number of inpatient days was 250,426 and there were 36,654 hospital admissions (dorsalgia and disc disorders representing 70%). A total of 100,000 magnetic resonance and 80,000 computerized tomography scans was adopted. Men had more inpatient days (138,215) than women (112,211). Overall, the inpatient/outpatient cost ratio was twice as high for men.

Conclusions We demonstrated that the direct costs of spinal disorders in Brazil in 2016 were high. We also found a substantial amount of financial resources spent on diagnostic imaging. This is relevant, as the routine use of diagnostic imaging for back pain is discouraged in international guidelines.

Keywords Cost of illness · Back pain · Costs and cost analysis · Hospital costs · Ambulatory care

Introduction

The functional and social impacts of spinal disorders on daily life and occupational activities have long been recognized (Gore et al. 2012). Chronic spinal disorders are one of the leading worldwide health problems, sustaining the highest rates of years lived with disability (GBD 2017) and prevalence ranging from 13 to 40% (Dean et al. 2014; Hoy et al. 2012). Moreover, spinal disorders can affect both men and women, within different age groups (Longworth et al. 2014; Muraki et al. 2014). The consequences of these conditions can also affect activities of daily living, work ability and quality of life (Weigl et al. 2008). Hence, poorer health conditions due to spinal disorders remain a concern for all members of society, such as workers and their families, employers and government (de Vroome et al. 2015). In this context, it is important to emphasize the increasing need of clinical decision-making based on scientific evidence, which supports the allocation of financial resources based on rational criteria (Ferraz 2015).

Cost-of-illness studies are useful and might help to determine the costs of the diagnosis and treatment of specific illness and to broaden the understanding of public health problems (Larg and Moss 2011). Evidence regarding the costs and economic burden of spinal disorders has been established in several countries since the 1990s (Asklof et al. 2014; Lambeek et al. 2011; Maniadakis and Gray 2000; van Tulder et al. 1995; Wenig et al. 2009). In Brazil, economic evaluations focusing on spinal disorders are still in its early stages, and there is scarce evidence on the use of financial resources directed to these diseases (Teles et al. 2016).

One study showed that the costs of ankylosing spondylitis in Brazil are considerable, though this was

Electronic supplementary material The online version of this article (https://doi.org/10.1007/s00038-018-1099-1) contains supplementary material, which is available to authorized users.

✉ Rodrigo Luiz Carregaro
rodrigocarregaro@unb.br

1 School of Physical Therapy, Universidade de Brasília (UnB), Campus UnB Ceilândia, Centro Metropolitano, conjunto A, lote 01, Brasília, DF CEP 72220-275, Brazil

2 Department of Health Sciences, Faculty of Science, Vrije Universiteit Amsterdam, Amsterdam, The Netherlands

3 School of Collective Health, Universidade de Brasília (UnB), Campus UnB Ceilândia, Brasília, Brazil

based on a cost analysis of a single outpatient clinic in 2005 (Torres et al. 2010). The Brazilian Social Security granted more than 1 million disability benefits to private sector workers in 2008, mostly due to spinal disorders (Vieira et al. 2011). Likewise, pension benefits due to spinal disorders covered approximately 12 million work days lost in 2007 (Meziat and Silva 2011). Moreover, it was estimated that costs of spinal surgeries had increased by 540%, in Brazil, in the past 20 years (Teles et al. 2016). It is well known that the Brazilian society is affected by the high prevalence of spinal disorders (Nascimento and Costa 2015; Zanuto et al. 2015). These findings were also corroborated by the Global Burden of Diseases Study, with recent data demonstrating that spinal disorders were one of the major components of worldwide disability, including Brazil (GBD 2017).

Nonetheless, gaps remain in our understanding of the major cost components of spinal disorders within the public healthcare system, which warrants further cost-of-illness studies with representative data. Therefore, the aim of the present study is to estimate the direct healthcare costs of spinal disorders in Brazil, over the year of 2016.

Methods

Study design

This is a prevalence-based cost-of-illness study with a top-down approach. We adopted the perspective of the public healthcare system. The study was approved by the Institutional Research Ethics Committee (Protocol n. 1.969.372; 16/03/2017).

We used the following International Classification of Diseases 10th revision (ICD-10) codes related to spinal disorders: M40 (kyphosis and lordosis); M41 (scoliosis); M42 (spinal osteochondrosis); M43 (other deforming dorsopathies); M45 (ankylosing spondylitis); M46 (other inflammatory spondylopathies); M47 (spondylosis); M48 (other spondylopathies); M49 (spondylopathies in diseases classified elsewhere); M50 (cervical disc disorders); M51 (other intervertebral disc disorders); M53 (other dorsopathies); M54 (dorsalgia); M96 (postprocedural musculoskeletal disorders, not elsewhere classified); M99 (biomechanical lesions, not elsewhere classified).

Data sources

Costs related to hospital inpatient and outpatient care were obtained (in Brazilian Reais—R$) from the Brazilian Health Ministry's Hospital Information System (SIH) and Outpatient Information System (SIA), respectively. The Hospital Information System contains all records of inpatient care which are processed and sent to the Ministry of Health and included in a National Database. The Outpatient Information System includes all outpatient care by public and private providers contracted by the Brazilian Public Health System (SUS). For that purpose, the Brazilian Healthcare System adopts a reference registered in the Table Management System of Procedures, Medical Drugs, Orthotics, Prosthetics and Special Materials of the Brazilian Health System (SIGTAP). Both systems are intended for the registration, control and processing of all healthcare provided, with an accounting and payment purpose (BRASIL 2017).

These systems present all procedures performed in the Brazilian Public Health System (SUS), which is financed by the Ministry of Health and covers approximately 75% of hospital and outpatient care in Brazil (Sussenbach et al. 2014). The systems use the ICD-10, which allows epidemiological analysis. The total number of hospital admissions in 2016 was 11,522,919 within a network of approximately [illegible]712 registered hospitals. In 2010, the Brazilian census (IBGE 2017) estimated 193,976,530 inhabitants, with a perspective of 206,081,432 inhabitants in 2016.

Inpatient costs

The following direct medical costs were obtained: hospitalization (i.e., daily rate; room fees; food; personal hygiene; bed support; hospital supplies; allied healthcare professional service costs; medications and diagnostic and therapeutic auxiliary services); medical professional service costs; intensive care unit (ICU) costs (including the use of all equipment for intensive care, technical teams and 24-h patient monitoring); companion daily stay (for each patient only one companion is allowed, and the amount includes adequate accommodation and provision of the main meals).

Outpatient costs

The Brazilian Outpatient Information System (SIA) accounts for all ambulatory services and procedures, such as medical and allied healthcare consultations, examinations, diagnostic imaging, clinical and surgical procedures, physiotherapy, acupuncture, rehabilitation and other procedures registered in the SIGTAP.

Data analysis

The inpatient and outpatient costs and procedures data are presented descriptively with tables and figures. The TABWIN software version 1.4.1 was used for extracting and processing data from the public health system. The

RETRACTED ARTICLE

outpatient costs were discriminated and reported separately for each category (clinical, surgical, diagnosis and, orthosis and prosthetics). For the inpatient care, as all costs are consolidated within the hospital system, the discrimination was not possible, and only the resource use (quantity) was presented for each category.

Age groups were classified as follows: < 1, 1–4, 5–11, 12–18, 19–28, 29–38, 39–48, 49–58, 59–68, 69–78 years and more than 79 years.

The population of each age group was based on the available information of the Brazilian population census of 2010. These data were used for the calculation of the ratio between the male population divided by the female population (ratio *M*/*F*). Additionally, the inpatient to outpatient costs ratio (*I*/*O* ratio) and the average cost per hospital admission (in US$; Currency—Dec 07, 2017: US$ 1 = R$ 3.2348) was calculated for each age group.

The total cost ratio (TCR) considered the total direct costs (in US$) spent per 1000 people, according to the following equation:

$$\text{TCR: } \frac{\text{Inpatient} + \text{Outpatient costs (US\$)}}{\text{Population (age groups)}} \times 10^3.$$

Results

In 2016, the Brazilian public healthcare system spent approximately US$ 714 million on spinal disorders and the inpatient costs represented 58% of the total direct costs (Table 1). The geographic distribution of these costs in Brazilian states is given in Online Resource Figure 1. Both inpatient and outpatient costs were more concentrated in the southeast and south regions.

The total number of inpatient days due to spinal disorders in 2016 was 250,426 and the number of hospital admissions was 36,654. Dorsalgia and intervertebral disc disorders represented approximately 70% of all hospital admissions in 2016 (Table 1).

Arthrodesis surgeries and treatment of complications were most frequently reported as the main reason for hospital admission (115,148 and 97,042 procedures, respectively—additional data are given in Online Resource Table [illegible]). Approximately, US$ 10 million was spent on diagnostic imaging (additional data are given in Online Resource Table 2), mostly due to magnetic resonance (MRI), computed tomography (CT) and ultrasonography (US). Specifically, around 36,000 MRI scans were used for low back pain with or without sciatica and 30,000 CT scans for low back pain only. Additionally, physiotherapy interventions were predominantly reported in the outpatient context, which represented approximately US$ 10 million (14% of the direct costs—Table 2).

The number of hospital admissions in 2016 due to spinal disorders between men and women was similar, except between the ages of 29–38 years, in which a slightly higher percentage (less than 2%) of admissions was found for men (Fig. 1). Additionally, men's admissions to hospitals accounted for a higher cost between the ages of 19–48 years, compared to women. Contrarily, the costs of outpatient care were higher for women, with a higher amount between the ages of 39–68 years compared to men (Fig. 2).

Table 3 presents the inpatient days and costs ratios among men and women during inpatient and outpatient care. Overall, men had more days of hospitalization compared to women between the ages of 19–68 years. The ratio between inpatient and outpatient costs was twice as high for men compared to women between the ages of 19–68. Likewise, the average cost per hospital admission tended to be higher for men.

Discussion

The aim of our study was to estimate the direct costs of spinal disorders in Brazil, from the perspective of the public healthcare system over 2016. This is the first study that investigated the costs related to spinal disorders using national data covering all of Brazil. We demonstrated that dorsalgia and intervertebral disc disorders accounted for the highest percentage of the direct costs. Moreover, approximately 14% of the direct costs were attributed to the routine use of diagnostic imaging. Men in the economically active age group (19–68 years of age) had slightly higher inpatient costs, while women had higher outpatient costs. Physiotherapy covered the largest part of outpatient care, accounting for around 14% of the direct costs.

We showed that in 2016, the Brazilian public healthcare system spent approximately US$ 72 million on spinal disorders, of which 58% were attributed to inpatient care. This finding is of utmost importance and might help to improve our understanding of the burden to the Brazilian society, considering that there is a high prevalence and well-known impacts of these conditions in different populational groups (Depintor et al. 2016; Nascimento and Costa 2015). Even though the comparison with international studies is difficult, we found similar costs components to those of other countries (Lambeek et al. 2011; Maniadakis and Gray 2000; Walker et al. 2003; Wenig et al. 2009). However, caution is suggested since the costs in Brazil were lower than in developed countries. For instance, similar cost-of-illness studies reported direct costs of approximately € 400 million in the Netherlands (Lambeek et al. 2011) and £ 1600 million in the United

RETRACTED ARTICLE

RETRACTED ARTICLE

Table 1 Direct healthcare costs of inpatient (hospital) and outpatient (ambulatory) care for spinal disorders in 2016

	Inpatient						Outpatient costs
	Admissions	Hc	Pc	ICUc	CSc	Inpatient costs	
Kyphosis and lordosis	52	120,226	15,574	17,551	511.93	153,864	231,400
Scoliosis	842	3,381,910	293,811	196,923	7636	3,880,281	821,996
Spinal osteochondrosis	51	30,822	7366	1970	405	40,565	19,566
Other deforming dorsopathies	539	1,048,272	127,881	46,230	3507	1,225,891	[illegible]
Ankylosing spondylitis	294	134,998	17,845	8651	1311	162,804	[illegible]
Other inflammatory spondylopathies	766	871,141	146,084	84,697	9210	[illegible]	100,336
Spondylosis	1060	605,648	108,023	49,137	10,666	[illegible]	444,366
Other spondylopathies	527	672,858	113,070	62,543	3455	851,926	188,984
Spondylopathies classified elsewhere	97	100,726	15,233	2775	1395	[illegible]	511,099
Cervical disc disorders	2202	2,624,601	439,776	279,981	[illegible]	[illegible]	928,829
Other intervertebral disc disorders	12,201	19,205,532	3,208,300	796,688	[illegible]	[illegible]	4,149,866
Other dorsopathies	156	191,246	32,275	28,581	[illegible]	253,053	329,571
Dorsalgia	13,143	1,771,210	276,105	57,943	[illegible]	2,155,637	18,472,097
Postprocedural musculoskeletal disorders	4075	2,662,950	391,280	219,632	32,383	3,306,245	1,160,103
Biomechanical lesions	649	504,441	96,921	[illegible]	2720	628,917	2,388,433
Total	36,654	33,926,584	5,289,547	[illegible]	199,993	41,294,262	30,119,656
Total direct costs in 2016 (US$)							71,413,918

Values are presented in US dollars. Currency: US$ 1 = R$ 3.2348

Hc hospital costs, *Pc* professional costs, *ICUc* intensive care unit costs, *CSc* companion stay costs

Kingdom (Maniadakis and Gray 2000). Likewise, Torres et al. (2010) demonstrated a lower cost for the treatment of ankylosing spondylitis in Brazil compared [illegible] other countries. The authors (Torres et al. 2010) highlighted methodological differences and a low per capita spending on healthcare in Brazil. Our findings could also be explained by healthcare systems operational differences (Ferraz 2015), discrepancies between perspectives and different cost components being included (Lambeek et al. 2011; Larg and Moss 20[illegible]).

Notwithstanding, some interesting comparisons are possible. We found an overall inpatient to outpatient cost ratio of 1.4, which could be interpreted as a relative equilibrium between inpatient and outpatient care and a fair amount of investment in outpatient facilities (Adam and Evans [illegible]). Data from previous studies are heterogeneous, with inpatient to outpatient ratios ranging from approximately 2 to 19 (Adam and Evans 2006; Lambeek et al. 2011; Maniadakis and Gray 2000), which could be attributable to differences in gross domestic product per capita, type of insurance (public or private), occupancy rate and hospital sizes within countries (Adam and Evans 2006). Nevertheless, it is recognized that outpatient care has advantages, such as to use hospital capacity for more complex patients and reduce costs, making it possible to treat more patients within the limited financial resources (Vitikainen et al. 2010). Outpatient services could help avoiding hospitalization-related costs, shorter operation times and lower complication rates of spine surgical procedures (Ahn et al. 2016). Therefore, the 2016s inpatient to outpatient cost ratio found in Brazil was interesting and warrants further investigations, to elucidate the trend pattern over a wider period of time. We would suggest that future studies investigate the impact of outpatient services on the efficiency and costs (Vitikainen et al. 2010) of inpatient spinal care in Brazil.

We found that dorsalgia and intervertebral disc disorders represented approximately 70% of all hospital admissions and 60% of the direct costs. This was expected, as previous Brazilian studies demonstrated a sustained and high prevalence of spinal disorders throughout the years (Depintor et al. 2016; Fernandes and Carvalho 2000; Zanuto et al. 2015). Additionally, our findings are similar to other countries and support the worldwide health burden due to spinal disorders (Asklof et al. 2014; Lambeek et al. 2011; Wenig et al. 2009). We also found that men aged between 19 and 68 years had slightly more inpatient days and more hospital admissions compared to women. There were fewer men than women in these age groups (ratio *M*/*F*), indicating that the relative cost for men was higher. This finding may be explained by epidemiological and socioeconomic aspects. For instance, the number of

RETRACTED ARTICLE

Table 2 Most common procedures and services adopted during outpatient care in 2016

Nature of procedure	Quantity	Most used	Quantity	Costs (US$)
Diagnostic	270,418	Magnetic resonance imaging: lumbosacral spine	82,494	6,897,010
		Computed tomography: lumbosacral spine	68,328	2,138,933
		Magnetic resonance imaging: cervical spine	23,177	1,935,270
		Computed tomography: cervical spine	21,728	583,3012
		Evaluation of respiratory function	721[illegible]	[illegible]22,295
Total diagnostic			202,939	11[illegible]54,515
Clinical	10,087,558	Physiotherapy: motor changes	8[illegible]87,766	[illegible]12,619,325
		Physiotherapy: pre- and post-surgery in musculoskeletal dysfunctions	1,04[illegible]36	2,061,215
		Multiprofessional/multidisciplinary team: rehabilitation of physical dysfunctions	191,308	1,162,537
		Physiotherapy: neuromusculoskeletal disorders without complications	45,066	65,061
		Clinical consultation of health profes[illegible]	21,568	42,005
Total clinical			9,992,844	15,950,143
Surgery	2296	Curative (with or without deb[illegible]dem[illegible]nt)	734	7352
		Joint manipulation (osteomusc[illegible] surgery)	665	5842
		Wound suture	321	2298
		Local anesthesia	320	2203
		Sedation	235	1101
Total surgery			2275	18,796
Orthoses, prostheses and special materials	3854	Brace: Pu[illegible] (hi[illegible]	1159	57,183
		Brace: Pu[illegible] (low)	986	59,438
		Brace: Milwa[illegible]	573	161,194
		[illegible]: [illegible]ost[illegible]n	311	57,541
		Brac[illegible] Philadelphia (immobilization of the cervical spine)	207	5106
Total orthoses, prostheses and special materials			3236	340,463
Total amount (in 2016)	12,[illegible]2,406	Total	10,179,726	27,886,212

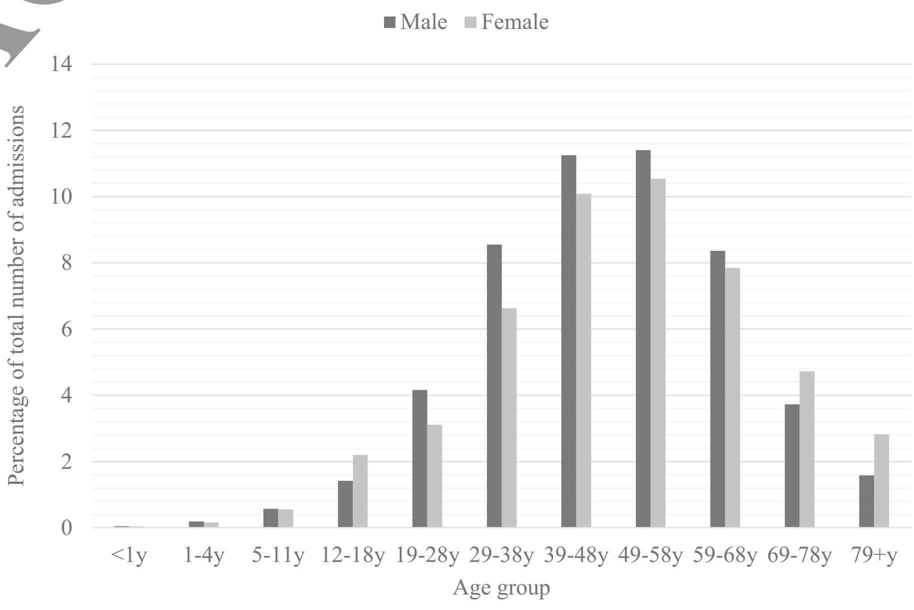

Fig. 1 Inpatient admissions among men and women, stratified by age groups (values are presented in % of the total number of admissions)

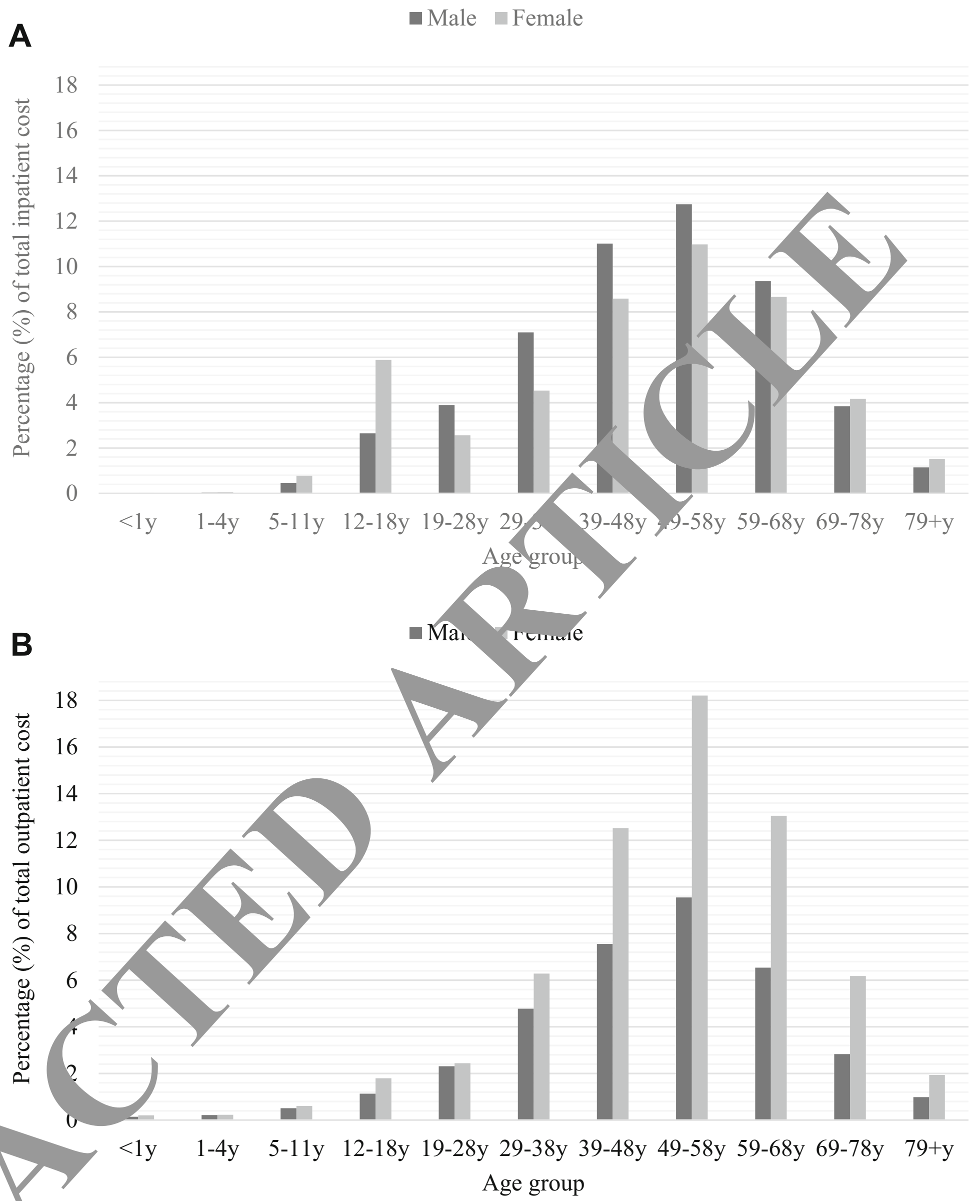

Fig. 2 Distribution of **a** inpatient (hospital) and **b** outpatient (ambulatory) direct costs among men and women, stratified by age groups (values are presented in % of the total cost)

RETRACTED ARTICLE

disability benefits claimed and the number of days off work due to back pain were higher for men in Brazil (Meziat and Silva 2011; Vieira et al. 2011). Also, it is usually assumed that men seek health assistance later, and with a worsened clinical condition (Galdas et al. 2005). Thus, it might be speculated that men needed more complex and costly actions during inpatient care. It is worth noting that inpatient and outpatient costs were higher in the south and southeast regions, which are more developed, industrialized and densely populated. Therefore, as the most affected age group was in the economically active range, the impacts of inherent risk factors such as increased occupational physical demand (Bevan 2015) might also explain the present costs.

Procedures adopted to justify hospital admissions were mostly the treatment of complications after surgical or clinical procedures, surgery (such as arthrodesis/vertebral fusion and discectomy), and drug treatment for severe pain. The employment of more complex interventions such as vertebral fusion is increasing with associated increase in costs and surgical complications (Balagué et al. 2012). There is a current debate regarding the efficacy of operative procedures in back pain (Koes et al. 2006), and systematic reviews reached conclusions towards limited or insufficient evidence of spinal surgery on clinical outcomes for lumbar spinal stenosis (Zaina et al. 2016) and lumbar spondylosis (Gibson and Waddell 2005). Additionally, a recent systematic review did not identify any evidence comparing surgical to non-surgical interventions for scoliosis with

Table 3 Values of inpatient days and cost ratios among men and women, stratified by age groups

	Age groups (years)										
	< 1	1–4	5–11	12–18	19–28	29–38	39–48	49–58	59–68	69–78	> 79
**Ratio M/F**	103	104	104	102	99	96	94	90	87	78	63
**Inpatient days**											
Men	171	401	1196	4113	11,083	19,954	27,193	31,107	26,679	11,648	4670
Women	105	349	1418	5462	7078	13,169	20,056	23,491	19,667	[illegible]	8212
**TCR**											
Men	35.4	14.1	29.4	111.9	116.2	286.9	540.8	888.3	[illegible]	845.1	651.6
Women	52.7	16.1	45.3	236.1	90.3	238.5	548.1	997.0	1187.8	972.4	645.5
**I/O ratio**											
Men	0.2	0.2	1.2	3.0	2.2	1.9	1.9	1.7	1.9	1.8	1.5
Women	0.2	0.3	1.7	4.3	1.4	0.9	0.9	0.8	[illegible]	0.9	1.0
**C/A ratio**											
Men	503	211	842	2002	1001	890	1050	1198	1200	1105	780
Women	834	340	1515	2870	882	734	913	[illegible]	1183	945	575

Ratio M/F ratio between the male population divided by the female population (values presented as quantity of men for 100 women), *TCR* (*total cost ratio*) total direct costs (inpatient + outpatient, in US$) per 1000 people, *I/O ratio* ratio between inpatient (*I*) and outpatient (*O*) costs, *C/A ratio* average cost per hospital admission (in US$)

severe curvature (Bettany-Saltikov et al. 2015). In the context of conservative treatments, physical therapy interventions were widely employed, but mainly in the outpatient setting, totaling approximately 10 million sessions in 2016. Physical therapy had a significant share of the direct costs (approximately 14%). Our findings are similar to a systematic review that demonstrated physical therapy as responsible for the highest percentage attributed to direct costs, with an average of 17% (Dagenais et al. 2008). This finding is interesting because physical therapy interventions focused on spinal disorders are widely recommended by international clinical guidelines (NICE 2016, 2017; Stochkendahl et al. 2018). Interventions such as exercise therapies are considered beneficial and were supported by low (Stochkendahl et al. 2018), moderate (NICE 2016) and strong (Delitto et al. 2012) evidence for the improvement of outcomes related to quality of life, pain and disability. Furthermore, though evidence-quality of structured exercise for spondyloarthritis was considered low, it was deemed to be useful for relevant outcomes such as fatigue, stiffness and joint mobility (NICE 2017). Nonetheless, future studies are warranted that evaluate whether conservative interventions are being implemented or whether implementation could be improved. It would also be important to analyze if conservative interventions are cost-saving and could reduce the number of spinal surgeries.

One interesting result was the high amount of routine diagnostic imaging adopted in 2016, which represented roughly 14% of the direct costs. It is worth mentioning that the costs are an underestimation of the real costs, as diagnostic imaging is also included in the costs of inpatient care. Nevertheless, our findings were higher compared to those reported by other studies, ranging from approximately 1 to 7% of the direct costs (Dagenais et al. 2008; Lambeek et al. 2011; Wieser et al. 2011). This is relevant and comprises decision-making implications, as international clinical guidelines rather discourage the frequent use of diagnostic imaging (NICE 2016). A considerable number of MRI and CT scans were used for low back pain or sciatica. It is important to note that the information included in our study does not allow inferences on the exact decision-making flow and patient context which determined the use of diagnostic imaging. Notwithstanding, the National Institute for Health and Care Excellence (NICE) guideline (NICE 2016) reported that there is no clear benefit for imaging all individuals with low back pain or sciatica. As a recommendation, the NICE guideline suggests alternative diagnoses, particularly in light of new or altered symptoms. Additionally, imaging should only be carried out in the presence of red flags and/or if the procedure will considerably change the management (for example, if epidural or spinal surgery is being considered), and not in response to a diagnostic uncertainty (Balagué et al. 2012; Koes et al. 2006; NICE 2016). Therefore, it is suggested that specific actions might be adopted to assess the decision-making process for using diagnostic imaging in back pain patients in Brazil, in light of up-to-date international guidelines.

We were not able to include the indirect costs in 2016, as the information was not available in the Brazilian Social Security System during the writing of this paper. However, we verified that in 2016, the Social Security System

RETRACTED ARTICLE

granted approximately 205,000 benefits due to spinal disorders, considering the same ICD-10 classifications adopted in this study. Including the costs of productivity losses would lead to much higher total costs and show a higher burden to the Brazilian society.

Our study has some limitations. First, we might have underestimated the direct costs related to spinal disorders, as the Brazilian's Hospital and Outpatient System covers approximately 75% of all healthcare services, though this is a representative sample. Second, inherent limitations to cost-of-illness studies should also be considered. For instance, the poor reliability of specific diagnostics (ICD-10 categories) and problems related to the diagnosis during hospital admission due to the lack of information in the patient's medical records (Bittencourt et al. 2006) might have influenced our findings. Third, as the private care and health insurances were not included in the present study, the direct costs of spinal disorders in Brazil would be higher.

Conclusion

Our study demonstrated that the direct healthcare costs of spinal disorders in the Brazilian public healthcare system in 2016 were very high. Dorsalgia and intervertebral disc disorders accounted for approximately 60% of these costs. A significant amount of financial resources was spent on diagnostic imaging, both during inpatient and outpatient care. This is an interesting finding, as international clinical guidelines recommend that the routine use of diagnostic imaging for back pain should be limited.

Acknowledgements To CAPES (Brazilian Federal Agency for Support and Evaluation of Graduate Education) for the Postdoctoral Research Abroad scholarship, process n. 88881.120102/2016-01.

Compliance with ethical standards

Conflict of interest The authors declare that they have no conflict of interest.

Ethical approval The Institutional Research Ethics Committee granted approval for the present study (Protocol n. 1.969.372; 16/03/2017). Informed consent was not needed, as public secondary data were used in the analysis.

Open Access This article is distributed under the terms of the Creative Commons Attribution 4.0 International License (http://creativecommons.org/licenses/by/4.0/), which permits unrestricted use, distribution, and reproduction in any medium, provided you give appropriate credit to the original author(s) and the source, provide a link to the Creative Commons license, and indicate if changes were made.

References

Adam T, Evans DB (2006) Determinants of variation in the cost of inpatient stays versus outpatient visits in hospitals: a multi-country analysis. Soc Sci Med 63:1700–1710. https://doi.org/10.1016/j.socscimed.2006.04.023

Ahn J, Bohl DD, Tabaraee E, Basques BA, Singh K (2016) Current trends in outpatient spine surgery. Clin Spine Surg 29:384–386

Asklof T, Kautiainen H, Jarvenpaa S, Haanpää M, Kiviranta I, Pohjolainen T (2014) Disability pensions due to spinal disorders. Spine 39:503–508

Balagué F, Mannion AF, Pellisé F, Cedraschi C (2012) Non-specific low back pain. Lancet 379:482–491. https://doi.org/10.1016/S0140-6736(11)60610-7

Bettany-Saltikov J et al (2015) Surgical versus non-surgical interventions in people with adolescent idiopathic scoliosis. Cochrane Database Syst Rev 4:CD010663. https://doi.org/10.1002/14651858.cd010663.pub2

Bevan S (2015) Economic impact of musculoskeletal disorders (MSDs) on work in Europe. Best Pract Res Clin Rheumatol 29:356–373

Bittencourt SA, Camacho LAB, Leal MC (2006) Hospital information systems and their application in public health. Cad Saude Publica 22:19–30

BRASIL (2017) DATASUS. Departamento de Informática do SUS. Ministério da Saúde. http://datasus.saude.gov.br/. Accessed 6 Dec 2017

Dagenais S, Caro J, Haldeman S (2008) A systematic review of low back pain cost of illness studies in the United States and internationally. Spine J 8:8–20. https://doi.org/10.1016/j.spinee.2007.10.005

de Vroome E, Uegaki K, van der Ploeg C, Treutlein D, Steenbeek R, de Weerd M, van den Bossche S (2015) Burden of sickness absence due to chronic disease in the Dutch workforce from 2007 to 2011. J Occup Rehabil 25:675–684. https://doi.org/10.1007/s10926-015-9575-4

Dean LE, Jones GT, MacDonald AG, Downham C, Sturrock RD, Macfarlane GJ (2014) Global prevalence of ankylosing spondylitis. Rheumatology (Oxf) 53:650–657. https://doi.org/10.1093/rheumatology/ket387

Delitto A et al (2012) Low back pain. Clinical Practice Guidelines Linked to the International Classification of Functioning, Disability, and Health from the Orthopaedic Section of the American Physical Therapy Association. J Orthop Sports Phys Ther 42:A1–57. https://doi.org/10.2519/jospt.2012.0301

Depintor J, Bracher E, Cabral D, Eluf-Neto J (2016) Prevalence of chronic spinal pain and identification of associated factors in a sample of the population of São Paulo, Brazil: cross-sectional study. Sao Paulo Med J 134:375–384. https://doi.org/10.1590/1516-3180.2016.0091310516

Fernandes RC, Carvalho FM (2000) Intervertebral disk disease among oil drilling workers. Cad Saude Publica 16:661–669

Ferraz M (2015) Health care: the challenge to deal with uncertainty and value judgment. Cost Eff Resour Alloc 13:8. https://doi.org/10.1186/s12962-015-0035-y

Galdas PM, Cheater F, Marshall P (2005) Men and health help-seeking behaviour: literature review. J Adv Nurs 49:616–623. https://doi.org/10.1111/j.1365-2648.2004.03331.x

GBD (2017) Global, regional, and national incidence, prevalence, and years lived with disability for 328 diseases and injuries for 195 countries, 1990–2016: a systematic analysis for the Global Burden of Disease Study 2016. Lancet 390:1211–1259. https://doi.org/10.1016/S0140-6736(17)32154-2

RETRACTED ARTICLE

Gibson JN, Waddell G (2005) Surgery for degenerative lumbar spondylosis. Cochrane Database Syst Rev 4:CD001352. https://doi.org/10.1002/14651858.cd001352.pub3

Gore M, Sadosky A, Stacey BR, Tai KS, Leslie D (2012) The burden of chronic low back pain: clinical comorbidities, treatment patterns, and health care costs in usual care settings. Spine 37:E668–E677. https://doi.org/10.1097/BRS.0b013e318241e5de

Hoy D et al (2012) A systematic review of the global prevalence of low back pain. Arthritis Rheum 64:2028–2037. https://doi.org/10.1002/art.34347

IBGE (2017) XII Censo Demográfico (2010). https://censo2010.ibge.gov.br/

Koes BW, van Tulder MW, Thomas S (2006) Diagnosis and treatment of low back pain. BMJ 332:1430–1434. https://doi.org/10.1136/bmj.332.7555.1430

Lambeek LC, van Tulder MW, Swinkels ICS, Koppes LLJ, Anema JR, van Mechelen W (2011) The trend in total cost of back pain in the Netherlands in the period 2002 to 2007. Spine 36:1050–1058

Larg A, Moss JR (2011) Cost-of-illness studies: a guide to critical evaluation. Pharmacoeconomics 29:653–671. https://doi.org/10.2165/11588380-000000000-00000

Longworth B, Fary R, Hopper D (2014) Prevalence and predictors of adolescent idiopathic scoliosis in adolescent ballet dancers. Arch Phys Med Rehabil 95:1725–1730. https://doi.org/10.1016/j.apmr.2014.02.027

Maniadakis N, Gray A (2000) The economic burden of back pain in the UK. Pain 84:95–103

Meziat N, Silva GAE (2011) Disability pension from back pain among social security beneficiaries, Brazil. Rev Saude Publ 45:494–502

Muraki S, Yoshimura N, Akune T, Tanaka S, Takahashi I, Fujiwara [illegible] (2014) Prevalence, incidence and progression of lumbar spondylosis by gender and age strata. Mod Rheumatol 24:657–661. https://doi.org/10.3109/14397595.2013.843761

Nascimento PR, Costa LO (2015) Low back pain prevalen[illegible] in Brazil: a systematic review. Cad Saude Public[illegible]1:1141–1[illegible]56. https://doi.org/10.1590/0102-311X00046114

NICE (2016) Low back pain and sciatica in over 16s: asses[illegible]ment and management. Assessment and non-invasive treatments vol NICE Guideline, No. 59. National Institu[illegible] for Health and Care Excellence (UK), London

NICE (2017) Spondyloarthritis in o[illegible]r 16s: diagnosis and management vol Guideline NG65. National [illegible]te for Health and Care Excellence (UK), London

Stochkendahl MJ et al (2018) National Clinical Guidelines for non-surgical treatment of patients with recent onset low back pain or lumbar radiculopathy. Eur Spine J 27:60–75. https://doi.org/10.1007/s00586-017-5099-2

Sussenbach SP, Silva EN, Pufal MA, Casagrande DS, Padoin AV, Mottin CC (2014) Systematic review of economic evaluation of laparotomy versus laparoscopy for patients submitted to Roux-en-Y gastric bypass. PLoS One 9:e99976. ht[illegible]ps://doi.org/10.1371/journal.pone.0099976

Teles AR, Righesso O, Gullo MC, Ghogawala Z, [illegible] A (2016) Perspective of value-based management of spi[illegible] disorders in Brazil. World Neurosurg 87:346–354. https://doi.o[illegible]g/10.1016/j.wneu.2015.11.047

Torres TM, Ferraz MB, Ciconelli RM (2010) [illegible]ource utilisation and cost of ankylosing spondylitis in Brazil. [illegible]in Exp Rheumatol 28:490–497

van Tulder MW, Koes BW, B[illegible]outer [illegible] (199[illegible]) A cost-of-illness study of back pain in the Neth[illegible]ands. [illegible] 62:233–240

Vieira ER, Albuquerque-Olivei[illegible] PR, Barbosa-Branco A (2011) Work disability b[illegible]fits due to musculoskeletal disorders among Brazilian private se[illegible]r workers. BMJ Open 1(1):e000003. https://doi.o[illegible]/10.1136/[illegible]jopen-2011-000003

Vitikainen K, Lin[illegible] M, Street A (2010) Substituting inpatient for outpatien[illegible]at is the impact on hospital costs and efficiency? [illegible] J Health Econ 11:395–404. https://doi.org/10.10[illegible]7/s10198-0[illegible]9-0211-0

Walker B[illegible] R, Grant WD (2003) Low back pain in Australian adul[illegible] the economic burden. Asia Pac J Public Health 15:79–87

Weigl M, Cieza A, Cantista P, Reinhardt JD, Stucki G (2008) Determinants of disability in chronic musculoskeletal health conditions: a literature review. Eur J Phys Rehabil Med 44:67–79

W[illegible]nig CM, Schmidt CO, Kohlmann T, Schweikert B (2009) Costs of back pain in Germany. Eur J Pain 13:280–286

Wieser S et al (2011) Cost of low back pain in Switzerland in 2005. Eur J Health Econ 12:455–467. https://doi.org/10.1007/s10198-010-0258-y

Zaina F, Tomkins-Lane C, Carragee E, Negrini S (2016) Surgical versus non-surgical treatment for lumbar spinal stenosis. Cochrane Database Syst Rev 1:CD010264. https://doi.org/10.1002/14651858.cd010264.pub2

Zanuto EA, Codogno JS, Christofaro DG, Vanderlei LC, Cardoso JR, Fernandes RA (2015) Prevalence of low back pain and associated factors in adults from a middle-size Brazilian city. Cienc Saude Coletiva 20:1575–1582. https://doi.org/10.1590/1413-81232015205.02162014

RETRACTED ARTICLE
